# P80, the HinT interacting membrane protein, is a secreted antigen of *Mycoplasma hominis*

**DOI:** 10.1186/1471-2180-4-46

**Published:** 2004-12-06

**Authors:** Miriam Hopfe, Ricarda Hoffmann, Birgit Henrich

**Affiliations:** 1 Institute of Medical Microbiology and Center for Biological and Medical Research, Heinrich-Heine-University, Moorenstrasse 5, 40225 Duesseldorf, Germany

## Abstract

**Background:**

Mycoplasmas are cell wall-less bacteria which encode a minimal set of proteins. In *Mycoplasma hominis*, the genes encoding the surface-localized membrane complex P60/P80 are in an operon with a gene encoding a cytoplasmic, nucleotide-binding protein with a characteristic Histidine triad motif (HinT). HinT is found in both procaryotes and eukaryotes and known to hydrolyze adenosine nucleotides in eukaryotes. Immuno-precipitation and BIACore analysis revealed an interaction between HinT and the P80 domain of the membrane complex. As the membrane anchored P80 carries an N-terminal uncleaved signal peptide we have proposed that the N-terminus extends into the cytoplasm and interacts with the cytosolic HinT.

**Results:**

Further characterization of P80 suggested that the 4.7 kDa signal peptide is protected from cleavage only in the membrane bound form. We found several proteins were released into the supernatant of a logarithmic phase mycoplasma culture, including P80, which was reduced in size by 10 kDa. Western blot analysis of recombinant P80 mutants expressed in *E. coli *and differing in the N-terminal region revealed that mutation of the +1 position of the mature protein (Asn to Pro) which is important for signal peptidase I recognition resulted in reduced P80 secretion. All other P80 variants were released into the supernatant, in general as a 74 kDa protein encompassing the helical part of P80. Incubation of *M. hominis *cells in phosphate buffered saline supplemented with divalent cations revealed that the release of mycoplasma proteins into the supernatant was inhibited by high concentrations of calciumions.

**Conclusions:**

Our model for secretion of the P80 protein of *M. hominis *implies a two-step process. In general the P80 protein is transported across the membrane and remains complexed to P60, surface-exposed and membrane anchored via the uncleaved signal sequence. Loss of the 4.7 kDa signal peptide seems to be a pre-requisite for P80 secretion, which is followed by a proteolytic process leading to a helical 74 kDa product. We propose that this novel form of two-step secretion is one of the solutions to a life with a reduced gene set.

## Background

The contact of a pathogenic bacterium with its eukaryotic host provokes a multitude of reactions. A prerequisite for successful infection with the host is the cytadhesion of the bacterium generally mediated by surface localized proteins [1]. Besides adhesion, pathogens like *Listeria*, *Yersinia *and even some of the mycoplasmas are able to invade the host cells [2-4]. An intracellular localization is obviously a privileged niche, as the bacteria are well protected from the immune system. Moreover, bacteria not only remain concealed, but have evolved strategies for an attack on the eukaryotic cell. In secreting virulence factors, such as antigenic or toxic proteins, bacteria can mislead the host immune response or damage the colonized tissue [5-7].

The large majority of exported proteins possess an N-terminal signal sequence [8]. Most signal sequences are recognized by the Sec-dependent protein translocation complex (translocase), which mediates membrane translocation of unfolded precursors [9]. The signal sequences of proteins predicted to be recognized by type I signal peptidases are composed of a short, positively charged amino-terminal region (n-region), a central hydrophobic region (h-region) and a more polar carboxyl region (c-region) containing the cleavage site [10]. The signal peptides present in pre-lipoproteins additionally contain a well-conserved lipobox with an invariant cysteine residue that is lipid-modified prior to precursor cleavage by signal peptidase II [11,12]. Cleavage of the signal peptide is not required for translocation of the proteins through the membrane, but is generally the final step in processing [13]. However, some precursors remain membrane bound because of an uncleaved hydrophobic signal peptide and diffuse laterally from the translocase [14].

In the last few years, computer programs such as PSORT-II, PSORT-B, ExProt and SignalP have been developed to facilitate the identification of putative secreted proteins [15-18]. Comparison of proteomes of Gram-negative bacteria, Gram-positive bacteria and Archaea using ExProt revealed that the fraction of putative secreted proteins ranged from 8% in the archaeal bacterium *Methanococcus jannaschii *to 37% in the mollicute *Mycoplasma pneumoniae *[17]. Analysis of the exported proteins of *Bacillus subtilis *found that only 50% of the secreted proteins were detected by genomic prediction, indicating that proteomic analyses of secreted proteins (the secretome) are necessary for a comprehensive definition of all secreted proteins [19].

Only a handful of mollicute genomes have been decoded, but no analyses of their secretomes have been conducted. A secreted protein, probably processed by the classical mechanism described above, has been characterized in the swine pathogen *M. hyopneumoniae*. P102 is encoded as a precursor protein carrying a type I signal sequence and is found exclusively in the extra-cellular milieu suggesting cleavage by signal peptidase I. The expression of secreted P102 is coupled to that of the surface-exposed cilium adhesin P97, which seems to represent a new variant of processed surface antigens. P97 is derived from a 126 kDa precursor protein by cleavage at amino acid residue (aa) 195. The cleaved 22 kDa N-terminal fragment, which carries an uncleaved type I signal sequence is found embedded in the membrane, in the cytoplasm and in a soluble form in the supernatant, whereas the mature P97, proposed to be membrane bound, is the target of complex proteolytic cleavage, which leads to the subsequent release of some fragments into the supernatant [20,21]. The lipoprotein MALP-404 of *M. fermentans *is a further example of a surface-localized protein undergoing proteolysis after reaching its target. Site-specific cleavage leads to the generation of the membrane bound immune-stimulatory lipopeptide MALP-2 and the release of the remainder (the RF fragment) in a soluble form into the supernatant [22].

In *M. hominis*, which is mainly found as a commensal in the urogenital tract, but has also been associated with human urogenital tract infections [23,24], two variants of a cell-surface protein exist, one a lipoprotein (P120) and a homologous P120' without a lipid anchor, but containing an uncleaved N-terminal signal sequence for type I signal peptidases [25]. Recently we identified a gene locus coding for a surface-localized protein complex composed of a 60 kDa lipoprotein (P60) tightly bound to an 80 kDa precursor protein (P80) with an uncleaved type I signal sequence [26]. We have proposed that the uncleaved N-terminus extends into the cytoplasm and thus mediates an interaction with the concomitantly expressed cytoplasmic HinT protein. HinT is found in prokaryotes and eukaryotes and in the latter is known to function as an adenosine nucleotidyl-hydrolase [27].

The data in this paper suggest that the signal peptidase I recognition site is protected in the membrane bound form of P80, but is accessible as the first step in the release of the helical part of P80 into the cell culture supernatant, a process which is accompanied by a decrease in size of 10 kDa.

## Results

**Figure 1:**
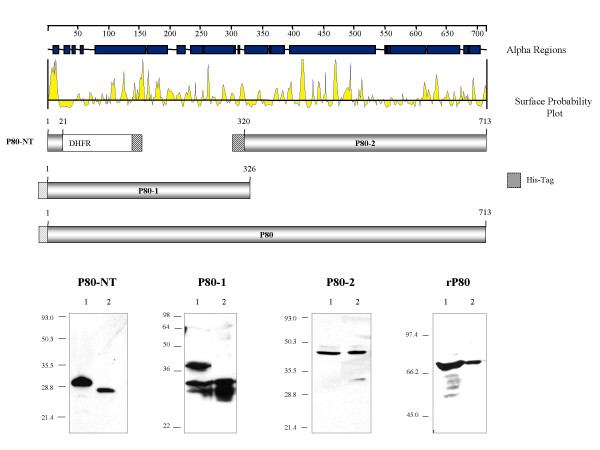
**Physical map and expression profile of recombinant P80 peptides**. The 713 AA polypeptide chain of the P80 precursor protein is schematically represented by an alignment of proposed α helical and surface localized regions. The expressed regions in the different P80 variants are shown below in gray flanked by the amino acid numbers corresponding to the position within the P80 precursor. The striped boxes represent the fused poly His tags, colored in black or gray, respectively, depending on their presence or absence after purification of the recombinant protein. P80-NT was expressed as a fusion with dihydrofolate reductase (DHFR). In the Western-blot analysis shown below, lysates (lane 1) and purified P80 variants (lane 2) have been immunostained with an anti-His_4_-antibody (P80-NT), or the P80-specific monoclonal antibodies NB12 (P80-1) or LF8 (P80-2, rP80).

To confirm our hypothesis that the P60/P80 membrane complex interacts with the cytoplasmic HinT via the uncleaved N-terminus of P80 that extends into the cytoplasm, we set out to express different fragments of the P80 protein to map the domain of P80 that interacted with HinT. As mycoplasmas use TGA as a tryptophan codon, rather than a stop codon as in *E. coli*, the TGA codons were mutated to TGG to allow expression. However, even though we mutated all TGA codons to TGG in the *hit*A gene, which encodes P80, we were not able to express and purify P80 peptides with an intact N-terminus. While P80-2, the C-terminal region of P80 from AA 320 to AA 713, was stably expressed in *E. coli*, P80-NT, the initial 21AA of the N-terminus, and P80-1, the N-terminal portion of P80 from AA 1 to AA 326, were degraded at the N-terminus during purification (Figure 1). Purification of the 2.6 kDa P80-NT fused to dihydrofolate reductase (DHFR) led to complete loss of the P80 region. As P80 NT did not contain the cleavage site of the signal peptidase I this proteolysis may be due to the presence of further processing signals within the first 21 AA of P80. The isolation of P80-1 revealed rapid degradation of the 40.7 kDa peptide with loss of 5, 6 and 8 kDa portions of the protein and loss of the poly His tag. Expression of the whole rP80 polypeptide chain (AA 1–713) resulted in a protein about 10 kDa smaller than expected, which lacked the poly His tag (depicted as a gray striped box in Figure 1). The conjecture that N-terminal degradation occurs was supported by Western blot analyses with anti-tetra His antibodies in which the purified forms of P80-1 and rP80 were not detectable (data not shown).

As conventional protease inhibitors had no effect on the P80 degradation (data not shown) and as type I signal peptidases share the unusual feature of being resistant to general inhibitors of the four other peptidase classes [10] we speculated that the degradation observed in the expressed rP80 may be in fact the result of physiological processing in *E. coli*.

### Analysis of the mechanism of rP80 processing

**Figure 2:**
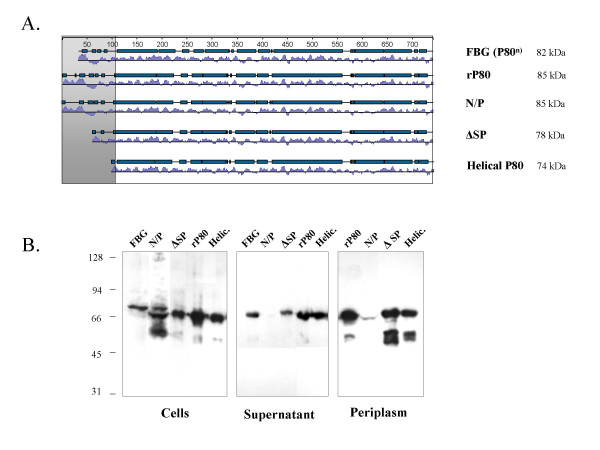
**Western blot analysis of the P80 variants**. A. The P80 variants are represented by alignments of their proposed α helical regions and hydropathy profiles. B. After cultivating the different P80 clones for 2–5 h in LB-Amp medium proteins in the supernatant (after 5 h cultivation), the periplasmic fraction and the cell pellet (after 2 h cultivation) were separated by 9.5% SDS-PAGE and subjected to Western blot analysis using the P80 specific monoclonal antibody LF8. The samples correspond to 10 μl of the recombinant P80 (**rP80**) culture, 1 ml of P80^Asn45Pro ^(**N/P**) culture, 10 μl of the helical P80 variant (**Helic.**) culture and 1 μl culture of the culture of the P80 variant without signal peptide (**ΔSP**). Samples from the culture supernatant were obtained from 30 μl (**rP80**), 0.8 ml (**N/P**), 0.2 ml (**Helic.**) and 60 μl (**ΔSP**) of media, and periplasmic proteins from 0.4 ml (**rP80**), 2 ml (**N/P**), 0.1 ml (**Helic.**) and 2 ml (**ΔSP**) of cultures. Additionally, lysate from 50 μl of culture and proteins from 250 μl of cell culture supernatant of *M. hominis *(strain **FBG**) were used. Marker, SeeBlue (Invitrogen, Germany).

In order to test the hypothesis that the degradation of rP80 is mediated by *E. coli *signal peptidase I, we characterized different mutants of the recombinant P80 protein according to their processing in *E. coli*. Using PCR, we constructed clones expressing the mature P80 protein without the 44 AA signal peptide (**ΔSP**) and an 89 AA N-terminal truncated P80 protein, which corresponds to the proposed helical, surface-localized part of P80 (**Helic.**). As the absence of proline at the +1 position of the mature protein is consistent with the observation that the SPase I of *E.coli *was inhibited by recombinant precursor proteins having a proline residue at this position [12], we generated a P80 precursor with an Asn to Pro mutation (**N/P**) at the (+1) position in the mature protein expecting a reduced or complete inhibition of signal peptide cleavage (Figure 2-A). Western-blot analysis of the heterologous expressed P80 variants in whole *E. coli *lysates revealed that a marked reduction of P80 processing was indeed observed for the (**N/P**) mutant, while the sizes of secreted P80 variants corresponded to that of the helical variant (Figure 2-B). Small amounts of rP80 precursor were only observed in the lysate after prolonged exposure in immunostaining with the P80-specific mAb LF8 (data not shown). All variants, with the exception of the N/P mutant, were transported across the inner and the outer membranes and were of nearly the same size as the secreted 74 kDa peptide of the P80 protein found in *M. hominis *(Fig. 2; FBG, supernatant). In the case of P80-(**ΔSP**) and P80-(**Helic.**), the two variants lacking signal sequences as translocation signals, this transport may be mediated by an alternative, Sec-independent export mechanism.

### P80 secretion in *Mycoplasma hominis*

The data suggest that the recombinant P80 processing in *E. coli *is signal peptidase I-mediated and therefore we investigated the protection of the signal peptidase I recognition site of P80 in *Mycoplasma hominis *[26]. Therefore, we analyzed the cellular proteins and the proteins in the culture supernatant in a mycoplasma culture in early-, mid- and stationary growth phases. To ensure that no viable cells were left in the supernatant we checked the separation efficiency by determining the titres of mycoplasmas in the supernatant and the original culture. The titres in the supernatants were 0.01% to 0.13% of those of the original cultures, demonstrating that the Western blot analysis would not be confounded by inclusion of cellular proteins in the supernatants (data not shown).

**Figure 3:**
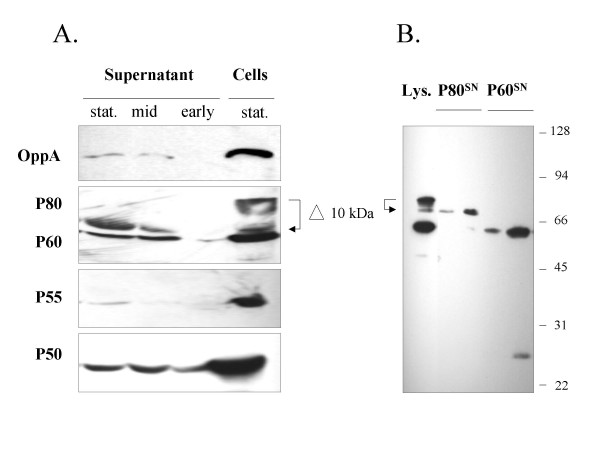
**Proteins released in the culture supernatant of *Mycoplasma hominis***. A. Western blot analysis of the proteins from 150 μl of supernatant and cells from 15 μl of culture, immunostained with the membrane protein specific monoclonal antibodies BG11 (OppA), LF8 (P80) and CG4 (P60), BG2 (P50), and the cytoplasmic protein specific monoclonal antibody AH10 (P55). B. Western blot analysis of P60 and P80 of *M. hominis *lysate (Lys.) from 15 μl of culture and of two different fractions of the purified P60 (P60^SN^) and P80 (P80^SN^) from the supernatant of a cell culture using sepharose-coupled antibodies LF8 (P80) or CG4 (P60).

As might be expected the stationary phase culture contained several proteins that might be released from lysed cells, including P50, P60, OppA and cytoplasmic proteins such as P55 (Figure 3-A). However, the same membrane proteins were found in the mid-logarithmic phase suggesting release of these proteins from living cells into the supernatant. Indeed, the amount of the cytoplasmic P55 was reduced in this culture suggesting that in mid-logarithmic phase few proteins from lysed cells are present. Lipoproteins, such as P50 and P60 were also found in the supernatant of a mycoplasma culture at the beginning of logarithmic growth (early), whereas OppA was absent. In general, immunostaining of P80 was weak in samples derived from the supernatant of a broth culture. This might be due to a masking of P80 by albumin, which was present in high concentrations in the culture medium and of similar size as the secreted P80 protein.

Interestingly, the P80 protein was the only one of the proteins analyzed that had a decreased size in the supernatant. To exclude the possibility that the reduction in size was simply a result of the large amount of albumin in the culture medium, we purified P80 from the supernatant of a culture in mid logarithmic growth using affinity chromatography. In addition we analyzed P60 as a control. As shown in Figure 3-B, the secreted P80 peptide was decreased in size by approximately 10 kDa in comparison to the cellular form, whereas P60 remained unaltered. A degree of processing of the P80 precursor was detectable, with several distinct P80 staining bands between 80 and 74 kDa, running through distinct steps of degradation as shown for the recombinant P80-1 peptide (see Figure 1).

**Figure 4:**
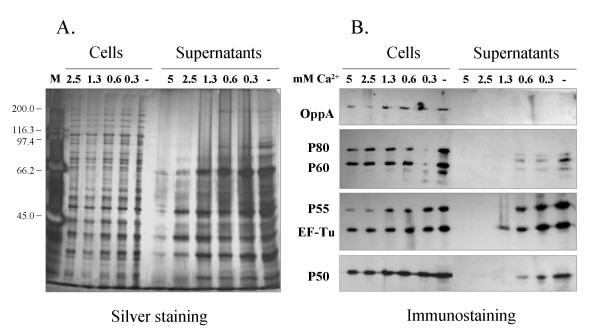
**Secreted antigens of *Mycoplasma hominis***. *M. hominis *cells from a mid-logarithmic phase culture were incubated for 1 h at 37°C in PBS containing 0 to 5 mM CaCl_2 _as indicated. Proteins in 0.4 ml of culture supernatants and cell pellets from 0.02 ml of cultures were separated in 9.5 % polyacylamide gels and silver stained (A.) or Western blotted (B.) using the monoclonal antibodies BG11 (OppA), LF8 (P80) and CG4 (P60), AH10 (P55), BG2 (P50) or KD2 (EF-Tu). The predominant band in the supernatant without calcium ions is recognized by the anti-P80 antibody LF8. Molecular Weight Marker: See Blue Plus 2 (Invitrogen, Karlsruhe, Germany).

As the activity of proteases often depends on the presence of divalent cations, we examined the secretion of mycoplasma proteins in the presence or absence of divalent cations. Mycoplasma cells were incubated for 1 h at 37°C in phosphate buffered saline (PBS) supplemented with up to 5 mM magnesium or calcium chloride, or chelating agents as EDTA and EGTA. The addition of divalent cations did not increase the processing of P80 (data not shown). As the presence of 5 mM calcium ions resulted in a complete disappearence of P80 from the supernatant we examined the release of mycoplasma proteins from cells following stepwise addition of calcium ions at concentrations of 0 to 5 mM (Figure 4). Silver staining of the cell and supernatant preparations revealed that only a few proteins were released in the supernatant whereas most proteins remained cell-embedded (Figure 4-A). Increasing the calcium chloride concentration to more that 1.3 mM led to a dramatic reduction in secretion. All the antigens analyzed, with the exception of OppA, were found in the supernatant, even the cytoplasmic proteins P55 and elongation factor Tu (EF-Tu) (Figure 4-B).

The cytoplasmic proteins that were detected in the supernatant were not released from lysed cells as the titres of the cultures remained the same throughout the experiment (data not shown). These findings are in accordance with those of Antelmann and coworkers, who found several cytoplasmic proteins, such as elongation factor G (EF-G) and arginase (RocF) as to be components of the secretome of *Bacillus subtilis *[19].

Increasing the concentration of calcium ions to more than 0.6 mM CaCl_2_inhibited a release of most mycoplasma proteins from the cells (Figure 4-B, lane 4). Of the proteins analyzed, only EF-Tu was found in the supernatant of a sample treated with 1.3 mM CaCl_2 _(Fig. 4-B, lane 3).

Thus the surface-localized P80 protein, normally complexed with the P60 lipoprotein in the membrane, appears to be released into the extra-cellular environment as a 74 kDa cleavage product. Our model of processing describes the initial cleavage of the 4.7 kDa signal peptide by a type I signal peptidase, followed by further degradation by unknown mechanisms, leading to a stable 74 kDa peptide.

## Discussion

The data presented here indicate that the membrane protein P80 of *Mycoplasma hominis *is a representative of a new group of proteins in the *Mollicutes*. P80 occurs as a precursor protein (complexed with the lipoprotein P60) anchored in the membrane and exposed on the surface, and is also released into the extra-cellular environment. As P80 and P60 are concomitantly expressed and both complexed in the membrane fraction it is likely that the soluble P80 variant is derived from the membrane bound precursor. Interestingly, the findings from the heterologous expression of different P80 mutants in *E. coli *suggest that release of P80 is initiated by cleavage of the N-terminal type I signal sequence, followed by amino-terminal proteolysis by an unknown mechanism, leading to a stable P80 peptide with a predicted helical structure in the supernatant.

As the genome of *M. genitalium*, the smallest known self-sufficient organism, lacks a gene for the type I signal peptidase [30] it was initially speculated that this enzyme may be absent in the *Mollicutes*, despite the fact that several proteins with type I-signal sequences had been identified [10,25]. However signal peptidase I genes have been found in the genomic sequences of *Mycoplasma gallisepticum *(MGA_1091) and *M. pulmonis *(MYPU_6300) [31,32].

The release of mycoplasmal proteins into the extra-cellular milieu may follow different pathways to those described previously for other bacteria. While the exclusive detection of P102 of *M. hyopneumoniae *in the extra-cellular matrix suggests that the intrinsic N-terminal type I signal peptide is immediately cleaved after translocation of the precursor through the membrane, as would be expected from current knowledge of the classical protein secretion pathway, the precursor proteins P80 and P120' of *M. hominis *are attached to the membrane without being processed or released into the supernatant [21,25,26].

The later release of a surface-localized peptide is described here for the first time. Davis and Wise described site-specific proteolysis of the lipid-anchored MALP-404 of *M. fermentans *that leads to the generation of the immune-stimulatory lipopeptide MALP-2 and showed that the residual RF peptide was soluble and was released into the supernatant [22]. Processing of the P97 cilium adhesin precursor, P126, of *M. hyopneumoniae *appears to be quite complex. The precursor protein P126, which carries a type I signal sequence with a cleavage site between AA 31 and AA 32, is predominantly cleaved between AA 194 and AA 195, suggesting that the SP-I signature may be essential only for the translocation of the whole precursor protein across the membrane. The cleavage products P22 and P97 both remain closely associated with the membrane. While P97 is generally subjected to further site-directed proteolysis, leading to the release of peptides into the extra-cellular milieu, further processing of P22 occurs to be strain dependent [21].

Release of proteins into the surroundings of a mycoplasma cell should lead to an immediate alteration of the cell surface architecture and, as most membrane proteins are targets for the host immune response [33,34], may also interfere with the host effector response. Type II secreted proteins appeared to be typically associated with non-invasive organisms colonizing mucosal surfaces, such as *Mycoplasma hominis*, and are considered to be required for the establishment of an infection at these sites. Recently in *Legionella pneumophila *a type II protein secretion system was characterized as a virulence factor linked to an intracellular infection [35].

P80 has significant similarity with two protein sequences, a hypothetical protein of *M. pulmonis *(MYPU_0060; gi15828477) the gene of which is followed by P60 (MYPU_0070) and HinT (MYPU_0080) gene homologues, and a rhoptry protein of *Plasmodium yoelii yoelii *(gi23481286). Rhoptry proteins are released during host cell invasion by apicomplexans [36].

As P80 interacts with HinT, a cytoplasmic protein found in all kingdoms, which in eukaryotes hydrolyses AMP derivatives and in yeasts functions as a regulator of an RNA polymerase II domain [27], a further scenario is imaginable: We do not know which factors promote the processing of the surface-exposed P80 protein. However, release of the helical P80 peptide is accompanied by the retention of the signal peptide I in the membrane. Some signal peptides have been found to leak back into the cell where they bind to proteins, such as the Ca^2+^-binding calmodulin [37], or are presented to the immune system [38], where they probably have a secondary signaling function distinct from their role in targeting [39]. Our results indicate that an increase in calcium ions prevents secretion of mycoplasma proteins. Thus, an extra- or intra- cellular stimulus – such as a local variation in the concentration of Ca^2+ ^ions – may activate the signal peptidase induced processing of the membrane bound P80 protein. This would lead to a soluble P80-helix and the membrane anchored signal peptide. After loss of the transmembrane spanning C-terminal amino acids or due to a leakage of the whole signal sequence (as described above) the signal peptide could reach the cytoplasm where an interaction with HinT may be a further step in modulating or promoting cellular processes such as growth or activation of gene expression, a process we are currently investigating.

## Conclusions

The data presented here clearly demonstrate that P80 is a secreted antigen of *Mycoplasma hominis*. This is, to our knowledge, the first description of secreted protein with a type I signal sequence that is stably embedded in the membrane as precursor protein and, as we propose as a model of secretion, is subsequently released from the membrane into the supernatant after signal peptidase I cleavage. As in other mycoplasmas several proteins have been shown to either possess an uncleaved signal sequence or to be further processed after anchoring in the membrane, this may be a common phenomenon in mycoplasmas. Because of their minimal coding capacity, mycoplasmas may have evolved a strategy to use secretory proteins in a dual role, as surface localized proteins in the membrane for cell surface architecture, and, in response to a change in the environment, as a soluble protein released from the cell surface.

## Methods

### Cloning and expression of rP80 and P80 mutants

The P80 protein encoding region (ACC Z29068) of *Mycoplasma hominis *strain FBG was amplified by PCR [26] using oligonucleotides (MWG Biotech, Ebersberg, Germany) that change the mycoplasma TGA tryptophan codon to TGG. Fragments were either directly cloned for the expression of distinct P80 regions or fused by SOE (splicing by overlap extension)-PCR [40]. To facilitate cloning of PCR products [41], restriction sites were inserted in the primer sequence without changing the amino acid sequence. P80 variants with mutations in the N-terminal part of P80 were generated by PCR by amplifying the region encoding the helical portion of P80 (**Helic., **AA 90–713), amplifying a fragment encoding the mature P80 polypeptide (**ΔSP**, AA 45–713), and mutating the asparagine codon at position +1 of the mature protein to a proline codon (**N/P**).

Amplicons were cut at the restriction sites within the primers and ligated in-frame into the expression vector pQE30 (Qiagen, Hilden, Germany). To express the P80 N-terminus (AA 1–21) as a fusion protein with dihydrofolate reductase and a poly His-tag, we ligated the respective *Rca*I/*Bgl*II restricted amplicon in frame into the *Nco*I/*Bgl*II restricted plasmid pQE60 and inserted the *Bam*HI/*Bgl*II restricted DHFR-fragment of pQE40 in the *Bgl*II site of the plasmid pQE60-P80 NT. Plasmids were propagated in DH5αF' for constitutive expression [41].

Cells from a mid-logarithmic phase culture in LB broth (Gibco BRL, Life Technologies Inc., Gaithersburg, Md.) containing ampicillin (100 μg/ml) were harvested by centrifugation (20,000 × *g*, 30 min, 4°C). In the secretion assay, the pelleted cells and supernatant, which was concentrated by lyophilizing, were subjected to Western blot analysis. The recombinant peptides P80-NT and P80-2 were purified by Ni-NTA chelation according to the manufacturer's protocol, while rP80 and P80-1 were purified with the Sepharose-coated P80-specific antibodies LF8 and NB12 as described by Henrich et al. [34].

### Sequence analysis

The analysis of DNA and protein sequences and the design of oligonucleotides were facilitated by use of the software package Lasergene (DNASTAR Inc. 1996, Madison, Wisc.). The integrity of the different plasmid sequences was confirmed by analysis on an ABI sequencer using the method of Sanger [42].

### Mycoplasma culture, osmotic lysis and secretion assays

*Mycoplasma hominis *strain FBG was cultivated in PPLO broth supplemented with arginine from frozen stocks of a mid-logarithmic phase broth culture as described previously [43]. The titer of the broth culture was determined by the measuring the number of color changing units (CCU) [34]. For protein secretion analyses, the mycoplasma cells from early- to mid-logarithmic phase cultures were harvested by centrifugation (20,000 × *g*, 10 min, 4°C), and the cell pellets and supernatants were subjected to Western blot analysis. Alternatively, cell pellets derived by low speed centrifugation (10,000 × *g*, 10 min, 4°C) were re-suspended in phosphate-buffered saline (PBS; 120 mM NaCl, 5 mM KCl, 20 mM Tris-HCl, pH 7.5) containing 5.0, 2.5, 1.25, 0.63, 0.31 or 0.00 mM CaCl_2 _and incubated at 37°C for 60 min. Soluble proteins were then separated from insoluble material by centrifugation (15,000 × g, 15 min, 4°C), and all samples analyzed by Silver staining [44] and Western blotting.

### Western blot analysis

Proteins were separated in 9.5% polyacrylamide gels [45], transferred to nitrocellulose (Schleicher and Schüll, Dassel, Germany) with a semidry blotting apparatus (Phase, Mölln, Germany) [46], and immunostained as described by Henrich et al. [34] using the monoclonal antibodies BG11 (anti-OppA), BG2 (anti-P50), LF8 (anti-P80), or CG4 (anti-P60), all of which are directed against membrane proteins, and AH10 (P55) and KD2 (EF-Tu), which are directed against proteins primarily located in the cytoplasm.

## Authors' contributions

RH began the characterization of antigens released into the supernatant of a *M. hominis *culture as part of her thesis. MH carried out the immunoassays and completed the secretion assays. BH carried out the molecular genetic studies, participated in the design of the study and drafted the manuscript. All authors have read and approved of the final manuscript.
